# Role of Kir4.1 Channels in Aminoglycoside-Induced Ototoxicity of Hair Cells

**DOI:** 10.1155/2023/4191999

**Published:** 2023-12-16

**Authors:** Jin Sil Choi, Ye Ji Ahn, SuHoon Lee, Dong Jun Park, JeongEun Park, Sun Mok Ha, Young Joon Seo

**Affiliations:** ^1^Research Institute of Hearing Enhancement, Yonsei University Wonju College of Medicine, Wonju, Republic of Korea; ^2^Department of Otorhinolaryngology, Yonsei University Wonju College of Medicine, Wonju, Republic of Korea

## Abstract

The Kir4.1 channel, an inwardly rectifying potassium ion (K^+^) channel, is located in the hair cells of the organ of Corti as well as the intermediate cells of the stria vascularis. The Kir4.1 channel has a crucial role in the generation of endolymphatic potential and maintenance of the resting membrane potential. However, the role and functions of the Kir4.1 channel in the progenitor remain undescribed. To observe the role of Kir4.1 in the progenitor treated with the one-shot ototoxic drugs (kanamycin and furosemide), we set the proper condition in culturing Immortomouse-derived HEI-OC1 cells to express the potassium-related channels well. And also, that was reproduced in mice experiments to show the important role of Kir4.1 in the survival of hair cells after treating the ototoxicity drugs. In our results, when kanamycin and furosemide drugs were cotreated with HEI-OC1 cells, the Kir4.1 channel did not change, but the expression levels of the NKCC1 cotransporter and KCNQ4 channel are decreased. This shows that inward and outward channels were blocked by the two drugs (kanamycin and furosemide). However, noteworthy here is that the expression level of Kir4.1 channel increased when kanamycin was treated alone. This shows that Kir4.1, an inwardly rectifying potassium channel, acts as an outward channel in place of the corresponding channel when the KCNQ4 channel, an outward channel, is blocked. These results suggest that the Kir4.1 channel has a role in maintaining K^+^ homeostasis in supporting cells, with K^+^ concentration compensator when the NKCC1 cotransporter and Kv7.4 (KCNQ4) channels are deficient.

## 1. Introduction

Ototoxicity is the cellular degeneration of cochlear and/or vestibular tissues. The cellular deterioration leads to functional deterioration [1]. Aminoglycoside antibiotics, loop diuretics, or antineoplastic agents including cisplatin have ototoxic properties. Ototoxicity is considered an otologic emergency because there is a low chance of recovery from functional damage when the appropriate treatment is not provided promptly. Cisplatin ototoxicity occurs in 23% to 50% in adults and up to 60% in children [2], while ototoxicity due to aminoglycosides and furosemide occurs in an estimated 25% and 6% to 7% of cases, respectively [3].

Aminoglycoside antibiotics disrupt cross-links between adjacent lipopolysaccharide molecules in the outer membrane for gram-negative bacteria and passively enter the periplasmic space [4]. Although little is known about the cellular and molecular mechanisms of drug ototoxicity, aminoglycosides can cause vestibular toxicity, which includes disequilibrium and dizziness, and cochlear toxicity, which can include hearing loss and/or tinnitus. Gentamicin enters the inner ear fluids from the strial capillaries through the strial marginal cells [5]. Both endocytosis and the mechanoelectrical transducer channel located at the top of hair cell stereocilia have been proposed to mediate the uptake of aminoglycosides into sensory hair cells [6–9]. The ototoxic drug affects the ion channels of the stria vascularis and causes hair cell death because it affects the endolymphatic potential [10]. Hair cell mechanoreceptors rely on gradients of potassium ion (K^+^) with a unique organization in the inner ear. K^+^ passively flows into the cell and circulates in the inner ear, where it may be important in maintaining the endolymphatic potential of cochlea [11].

Among the ion channels in the inner ear, K^+^ inwardly rectifying 4.1 (Kir4.1) channel is an important conduit for K^+^ to the endolymphatic space for generation of the endolymphatic potential. Kir4.1 is an important mechanism for the delivery of K^+^ to the K^+^-secretory strial marginal cells in the stria vascularis. The Kir4.1 channel is also present in hair cells, particularly in the basal side of the hair cell, where it may be important in the K^+^ transport of potassium [11, 12]. Weak Kir4.1 labeling of Deiters' cells surrounding the outer hair cell layer has been described [13, 14]. This specific location implies that Kir4.1 in the organ of Corti may also play a role in K^+^ absorption for the recycling of K^+^ in the cochlea [15].

The Kir4.1 channel mutations to human diseases are EAST syndrome (Bockenhauer et al., 2009) [16] and SeSAME syndrome (Scholl et al., 2009) [17] describing epilepsy, ataxia, sensorineural deafness, tubulopathy (EAST syndrome) and seizures, sensorineural deafness, ataxia mental retardation, and electrolyte imbalance (SeSAME syndrome) caused by mutations in KCNJ10 gene encoding Kir4.1 channels [16, 17]. Other syndromes are also directly related to Kir4.1 dysfunction. These are Rett's syndrome (Olsen et al., 2015; Nwaobi et al., 2016) [18, 19] and Alper's syndrome (Smith et al., 2023) [20]. There are diseases linked to the downregulation of Kir4.1 channels such as diabetes (Pannicke et al., 2006; Rivera-Aponte et al., 2015) [21, 22] and Huntington's disease (Proft and Weiss, 2014) [23].

In our previous study, kanamycin and furosemide have each been used in single application regimens in ototoxic drug-induced hearing loss animal models [24]. We thought that the combined use of kanamycin and furosemide could directly affect Kir4.1. We explored the importance of the Kir4.1 channel in this combination treatment using HEI-OC1 cells grown in a condition in which Kir4.1 is highly expressed. The cochlear cell line (progenitor HEI-OC1) cells are multipotent progenitors called “House Ear Institute-Organ of Corti 1 (HEI-OC1)” cells, which are used for in vitro screening of ototoxic drugs. These cells are common progenitors for auditory (receptor and sensory) cells and for supportive cells (Kalinec et al., 2016) that are susceptible for commonly used drugs such as cisplatin [25].

## 2. Materials and Methods

### 2.1. HEI-OC1 Cell Culture

HEI-OC1 cells provided by Kalinec et al., House Ear Institute (Los Angeles, CA, USA), were cultured in high-glucose Dulbecco's modified Eagle's medium (Gibco BRL, Gaithersburg, MD, USA) supplemented with 10% fetal bovine serum (Gibco BRL, Gaithersburg, MD, USA) and 50 U/ml gamma-interferon (Genzyme, Cambridge, MA, USA) without any antibiotics in a permissive condition (33°C, 10% CO_2_). For the nonpermissive condition, cells were first cultured in the permissive condition for a certain period and then transferred to 39°C, 5% CO_2_ [26]. Growth curves were performed to identify the growth patterns of cells in each condition. Cells cultured at 33°C in a 10% CO_2_ atmosphere were recorded on day 0 after cell counting before seeding. Cells were recovered daily from the cell dish and enumerated. This was done for up to 14 days. In the cells shifted to 39°C, incubation at 33°C was done for 5 days (which was determined to be optimal for differentiation in preliminary experiments), and cells were enumerated just prior to being shifted to the higher temperature condition. Following the shift, cells were enumerated daily for 14 days.

### 2.2. Preparation of Ototoxic Drugs

As explained above, HEI-OC1 cells were cultured in permissive conditions (33°C, 10% CO_2_) until day 5 and then transferred to the nonpermissive condition (39°C, 5% CO_2_).

Four drug treatment groups were used: untreated (control), kanamycin, kanamycin and furosemide (both from Cayman Chemical, Ann Arbor, MI, USA), and furosemide. In preliminary experiments, the CCK assay was performed as described next to confirm if the treatment concentration affected viability. Almost no cell death was observed with 1 mM kanamycin or 50 *μ*M furosemide. Subsequent experiments used 100 *μ*M kanamycin and 10 *μ*M furosemide to ensure no deleterious effects on cell viability. High-glucose DMEM supplemented with 0.5% FBS was used.

### 2.3. CCK Assay

After the treatment of HEI-OC1 cells with the aforementioned concentrations of kanamycin and furosemide, the EZ-3000 CCK assay (Dogen, Seoul, Korea) was performed. After culturing the cells for 4 days at 39°C, Trypsin-EDTA (Gibco BRL, Gaithersburg, MD, USA) was used to remove the cells, which were seeded (2 × 10^4^ cells/ml) in wells of a 96-well dish. The total volume of each well was 100 *μ*l, and there were five wells of each of the four aforementioned groups, for a total of 20 wells. After 24 hours, the medium in each well was replaced with the drug-containing medium. CCK was measured from 24 to 168 hours by the addition of 10 *μ*l CCK solution to each well, incubation for 1 hour, and measurement of the absorbance was measured to determine cytotoxicity.

### 2.4. Reverse Transcriptase Polymerase Chain Reaction (RT-PCR) and Quantitative Real-Time (qRT)-PCR

One milliliter of TRIZOL (Invitrogen, Carlsbad, CA, USA) was added to cells (5 × 10^6^ cells/ml), distributed by vortexing, and incubated for 5 minutes at room temperature (20°C-25°C). Two hundred microliters of chloroform was added, mixed by vortexing for at least 30 seconds, and incubated at room temperature for 10 minutes. The sample was recovered by centrifugation at 12,000 rpm for 15 minutes, and the supernatant was transferred to a new tube. An equal volume of 2-propanol was added and vortexed for at least 30 seconds. The sample was incubated at room temperature for 10 minutes and recovered by centrifugation at 11,000 rpm for 10 minutes. The supernatant was discarded, and the pellet was stored on ice. Distilled water (30-50 *μ*l) was added, and the RNA was dissolved at 55°C for 10 minutes using a heat block. The extracted RNA was quantified, and cDNA (TOYOBO, Osaka, Japan) was synthesized with 1 *μ*g of RNA. 4xDN was added and incubated at 37°C for 5 minutes. Reverse transcriptase (5x) was added and incubated at 37°C for 15 minutes followed by 98°C for 5 minutes. The synthesized cDNA was diluted 1/5 and used for PCR. To screen the Kir4.1 ion channels in HEI-OC1 cells, RT-PCR was performed. Template (4 *μ*l), primer (1 *μ*l), and Hot Taq DNA polymerase (10 *μ*l) (Komabiotech, Seoul, Korea) were added to 5 *μ*l of distilled water, and PCR was performed with one cycle of initial denaturation at 95°C for 15 minutes; 35 cycles of denaturation (94°C, 30 seconds), annealing (51-58°C, 30 seconds), and extension (72°C, 1 minutes); and one cycle of a final extension (72°C, 10 minutes). We confirmed Kir4.1, Na-K-2Cl cotransporter-1 (NKCC1), potassium voltage-gated channel subfamily Q member 4 (KCNQ4), Kcnmb1, ClcnkA, ClcnkB, Scnn1A, Scnn1G, Gja1, Gjb6, Slc26A4, Atp2B1, Atp6V0A4, and *β*-actin by RT-PCR [27] (Supplement Table 1). After PCR was completed, electrophoresis was performed for 15 minutes with a 2% agarose gel mixed with loading dye. The resolved band was confirmed using a Bioanalytical Imaging system. The RNA levels of Kir4.1, NKCC1, and KCNQ4 were confirmed by qRT-PCR by adding 14 *μ*l of template, 3.5 *μ*l of primer, and 17.5 *μ*l of SYBR (Applied Biosystems, Foster, CA, USA) to make a total volume of 35 *μ*l. Each well of a 384-well plate was loaded with 10 *μ*l. Each sample was loaded in triplicate. Real-time PCR was performed using one cycle of 95°C for 10 minutes; 40 cycles of 95°C for 15 seconds, 54°C for 1 minute, and 72°C for 30 seconds; and one final cycle of 95°C for 15 seconds, 60°C. After the PCR was completed, a graph was created using the GraphPad Prism program.

### 2.5. Western Blot Analysis

Pro-prep solution (200 *μ*l) (Intron Biotechnology, Seongnam, Korea) was added to a pellet-shaped cell and vortexed to create a homogenous suspension. Each sample was kept on ice for 30 minutes and centrifuged at 13,000 rpm for 20 minutes. The supernatant was transferred to a new tube. The extracted proteins were quantitated using the Bradford assay, and the defined quantity of total protein was used for a western blot assay. SDS-PAGE (10%) was performed at 0.02 A for 2.5 hours. The resolved proteins were transferred to a PVDF membrane at 100 V for 1 hour. After transfer, proteins were confirmed by the Ponceau S stain and blocked with 5% skim milk for 1 hour at room temperature. Primary antibodies were against Kir4.1, NKCC1 (ab59791; Abcam, Cambridge, UK), KCNQ4 (ab65797, Abcam, Cambridge, UK), and *β*-actin (Santa Cruz Biotechnology, Dallas, TX, USA). Each primary antibody was diluted 1 : 1,000 in 2.5% skim milk and incubated overnight at 4°C. The secondary antibody was diluted 1 : 10,000 in 1% skim milk and incubated for 1 hour at room temperature. After washing five times with 1x Tris-buffered saline Tween 20 (TBST) for 5 minutes, ECL solution (Millipore, USA) was added for 1 minute, and the band was confirmed with the ChemiDoc gel imaging system (Bio-Rad, Hercules, CA, USA).

### 2.6. Immunofluorescence

Immunofluorescence was determined in vitro and ex vivo. In vitro, cells were cultured at 33°C or 39°C for 3 or 7 days. Cells were then seeded (4 × 10^4^ cells/ml) in 4-well dishes. After culturing for 24 hours, each sample was washed with 1 ml PBS, and 500 *μ*l of 4% paraformaldehyde was added to each well and fixation proceeded for 1 hour. The paraformaldehyde was removed, and each sample was washed three times for 5 minutes each in PBS. After the final wash, 500 *μ*l of 0.1% Triton X-100 was added and incubated for 15 minutes at room temperature. Triton X-100 was removed, and samples were washed three times for 5 minutes each in PBS. The primary antibody (Kir4.1, APC-035; Alomone labs, Jerusalem, Israel) diluted 1 : 100 in 5% normal goat serum (NGS) was added, and the cells were incubated for 1 hour at room temperature. PBS (500 *μ*l) was then added to each well and washed three times (5 minutes per wash). The secondary antibody (goat anti-rabbit IgG H&L, ab150077; Abcam, Cambridge, UK) was diluted 1 : 200 in 5% normal goat serum (NGS) and incubated for 1 hour at room temperature. Each sample was then mounted using mounting solution and stained with 4′,6-diamidino-2-phenylindole (DAPI), and a cover glass was added. The solution was dried for 24 hours in a dark room and examined using confocal microscopy.

### 2.7. Small Interfering RNA (siRNA) Transfection

After culturing at permissive conditions of 33°C and 10% CO_2_, the cells were transferred to 39°C. After 2 days, the cells were detached with Trypsin-EDTA and seeded in a 60 mm dish at a density of 3 × 10^5^ cells/ml. After 24 hours, Kir4.1 siRNA (sc-42625, Santa Cruz Biotechnology, Dallas, TX, USA) was added (40 pmol). After 6 hours, 1 ml of medium containing 2X FBS was added to each well and incubated for 48 hours. The cells were subjected to PCR and western blotting as described above. When drug treatment (100 *μ*M kanamycin or 10 *μ*M furosemide) was performed within the Kir4.1 inhibited condition, the drug was added 48 hours after the siRNA treatment (39°C, day 5) and maintained for 24 hours.

### 2.8. Animal Experiments and Paraffin Section

The animal study protocol was approved by the Institutional Animal Care and Use Committee of Yonsei University, Korea (YWC-180703-1), and the procedures followed were in accordance with the institutional guidelines. Animals were purchased through the “Daehan Bio” company according to institutional guidelines. Animals were cared for at a temperature of 18-26°C, and the humidity was maintained at 40-60%.

In addition, the bedding was changed twice a week, and the cage and water bottle were sterilized before use.

To alleviate the suffering of animals through an experimental process, mice were anesthetized with a mixture of ketamine and Rompun.

In the drug-induced ototoxicity animal model, 550 mg/kg kanamycin and 130 mg/kg furosemide were injected as previously described [24]. Ototoxicity was evident in mice as decreased audiometry from the third day after the Auditory Brainstem Response test (data not shown). The mice exposed to kanamycin or furosemide were sacrificed on day 14. Cardiac perfusion was performed with 1× PBS (50 ml) and fixed with 50 ml of 4% paraformaldehyde. Only the cochlea was obtained from each mouse, and 4% PFA was added at 4°C overnight. Twenty-four hours later, the decalcification process was performed with Calci-Clear Rapid (Chayon Laboratories, INC, Seoul, Korea), followed by dehydration with 30% sucrose. The cochlea were paraffin embedded and sections were obtained at a thickness of 5 *μ*m. Sections were deparaffinized by dipping for 5 minutes in Neo-clean, and dehydration was performed using 100%, 95%, 90%, and 70% ethanol. The slides were washed with 1× PBS for 5 minutes, and immunofluorescence was performed as described above. In ex vivo samples, the cochlea were obtained from sacrificed C57BL/6 mice (6 weeks of age, male). After the bulla was removed from the cochlea, a hole was made in the apex portion of the cochlea to remove the bone. In the cochlear turn, the stria vascularis and Reissner membrane were removed to leave only the organ of Corti. The sensory epithelium was placed on a one-well slide dish and immunofluorescence was performed.

### 2.9. Explant Models

We performed a cochlear surface prep to check the surface of the cochlear. Mice were anesthetized with ketamine and Rompun to relieve pain. The anesthetized mouse was euthanized by performing cervical dislocation, and the cochlear was harvested.

Cochlear performed surface prep immediately after harvesting to keep it as fresh as possible. We used H buffer for cochlear surface prep, and H buffer was made with 1/10 HEPES (Gibco BRL, Cat. 15630080, Gaithersburg, MD, USA) and 10 mM HBSS (Stemcell, NC9162583, Vancouver, Canada) in D.W. After that, it was cultured for 24 hours in explant culture media, and the composition was DMEM F12 media (Gibco BRL, Cat. 11320033, Gaithersburg, MD, USA), 10% FBS (Gibco BRL, Gaithersburg, MD, USA), ampicillin 10 *u*g/mL, 1% N2 supplement (Gibco BRL, Cat. 17502048, Gaithersburg, MD, USA), and 1% B27 (Gibco BRL, Cat. 17504044, Gaithersburg, MD, USA).

### 2.10. Statistical Analysis

Statistical analysis was performed using SPSS statistical package version 21.0 (SPSS Inc., USA). Descriptive results of continuous variables are expressed as the mean ± standard deviation (SD) for normally distributed variables. Means were compared by 2-way analysis of variance. The level of statistical significance was set to 0.05.

## 3. Results

### 3.1. Screening of Ion Channels in HEI-OC1 Cells

**Figure 1:**
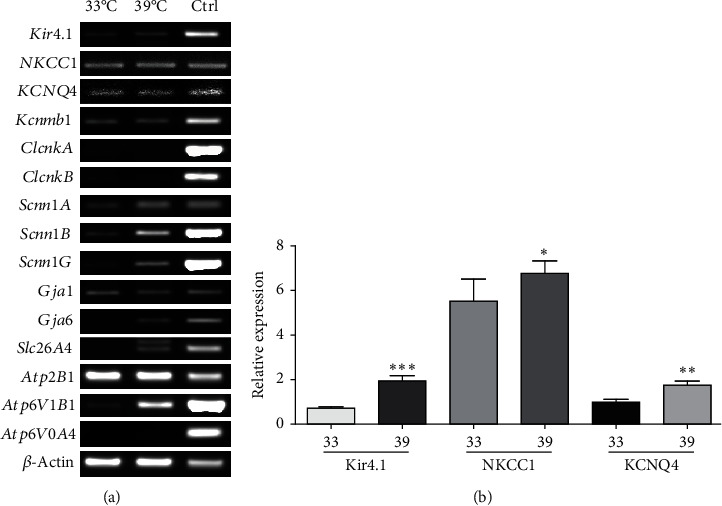
Screening of hair cell markers in HEI-OC1 cells and the expressions of the main potassium channels. HEI-OC1 cells were cultured at 33°C followed by 39°C, each for 5 days. Expression of marker genes in hair cells was confirmed and expression of main potassium channels was confirmed. (a) RT-PCR results of hair cell markers in HEI-OC1 cells. RT-PCR was performed on day 5 of incubation at 33°C and 39°C. Kidney tissue was used as a control. (b) Expression of marker genes and main potassium channels were confirmed in day 5 HEI-OC1 cells at 33°C and 39°C.

Ion channels are expressed in hair cells. HEI-OC1 is an immortalized organ of the Corti-derived epithelial cell line. In this study, we screened for representative ion channels in HEI-OC1 cells. Sixteen ion channels were identified by RT-PCR using the primer sequences summarized in Supplementary Table 1. The control used mouse kidney and compared the expression levels in permissive and nonpermissive conditions. Most of the channels were highly expressed at 39°C. In particular, the expression of the potassium-related channels Kir4.1, NKCC1, and KCNQ4 was increased in the differentiation conditions (Figure 1(a)). When the expression levels of the main potassium channels Kir4.1, NKCC1, and KCNQ4 were obtained by qRT-PCR, the expression of all three channels was greater in the nonpermissive condition than in the permissive condition (Figure 1(b)). Based on these results, the 39°C condition was used to achieve the increased expression of ion channels.

### 3.2. Changes in Expression of Kir4.1 in HEI-OC1 Cells at Different Conditions

**Figure 2:**
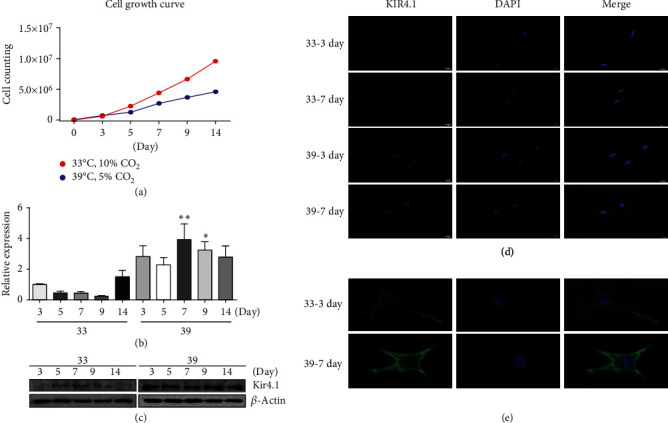
Characteristics of HEI-OC1 cells and the expression of Kir4.1. Cell growth curve and Kir4.1 expression levels were observed to determine the experimental condition in HEI-OC1 cells. (a) Cell growth curves were obtained at 33°C and 39°C for 14 days in HEI-OC1 cells. (b) The change in the expression level of Kir4.1 was confirmed by qRT-PCR. (c) Expression of Kir4.1 at 33°C and 39°C was confirmed at the protein level by western blot. (d, e) Immunofluorescence assay was performed to confirm the changes in the expression level of Kir4.1 on day 3 and day 7 of cells at 33°C and 39°C, respectively. Expression of Kir4.1 was enhanced at 39°C compared to 33°C. High magnification microscopy revealed that the expression was mainly at the cell membrane surface.

Growth curves of HEI-OC1 revealed that the permissive condition was significantly increased from day 5 compared to the nonpermissive condition. In the latter, proliferation was slightly prolonged to 14 days (Figure 2(a)). Kir4.1 was highly expressed at 39°C compared to 33°C, which was confirmed at the protein level as well as at the RNA level (Figures 2(b) and 2(c)). Immunofluorescence revealed that the expression was significantly increased at 39°C compared with 33°C, and the expression level was further increased at day 7 than at day 3 (Figure 2(d)). Microscopy examination revealed the increased expression of Kir4.1 throughout the cytoplasm (Figure 2(e)).

### 3.3. Role of Kir4.1 in HEI-OC1 Cells Treated with Kanamycin and/or Furosemide

**Figure 3:**
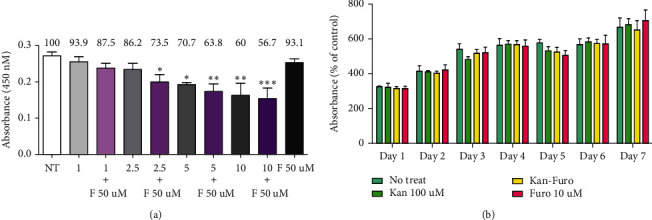
Concentration conditions of ototoxic drug treatment in HEI-OC1 cells and in the CCK viability assay. Drug concentrations that lead to cell death and cell damage were observed up to day 7 to establish the ototoxic drug dose. (a) Measurement of concentrations of kanamycin and furosemide to determine safe treatment dose. (b) CCK assays performed after 7 days with kanamycin 100 *μ*M and furosemide 10 *μ*M at 39°C and 5% CO_2_.

**Figure 4:**
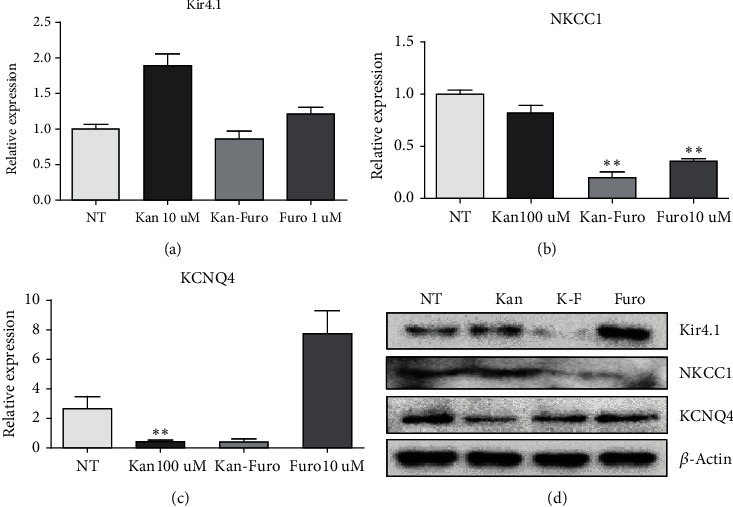
Association of potassium channels Kir4.1, NKCC1, and KCNQ4 in HEI-OC1 cells. Western blot and qRT-PCR results to determine the association of Kir4.1, NKCC1, and KCNQ4 in HEI-OC1 cells. (a) Treatment of HEI-OC1 cells with either kanamycin or furosemide increased the expression of Kir4.1. (b) When furosemide was treated in HEI-OC1 cells, NKCC1 was blocked, and kanamycin was not affected. (c) When kanamycin was treated in HEI-OC1 cells, KCNQ4 was blocked, and there was no specific effect upon furosemide treatment. (d) Changes in expression levels of potassium channels in ototoxic drug-treated HEI-OC1 cells confirmed by western blotting.

**Figure 5:**
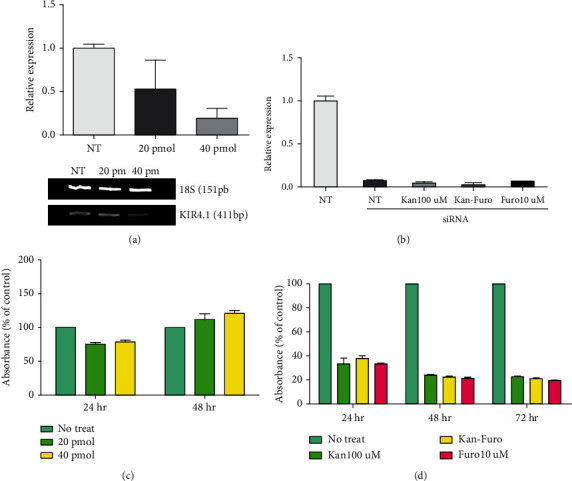
Changes in HEI-OC1 cells upon Kir4.1 channel inhibition. (a) qRT-PCR results of HEI-OC1 cells cultured 39°C for 3 days following Kir4.1 inhibition. (b) Expression of Kir4.1 with ototoxic drug treatment in HEI-OC1 cells cultured at 39°C for 3 days following siRNA transfection. (c) CCK assay of cell viability following transfection with Kir4.1 siRNA. (d) CCK assay results of Kir4.1 channel inhibition HEI-OC1 cells after 72 h of treatment with ototoxic drugs.

Ototoxic drugs affect the stria vascularis and the endolymphatic potential. To confirm whether the drugs could directly affect the ion channels of hair cells, the doses of the ototoxic drugs kanamycin and furosemide that were previously established in the mouse model were converted into treatments of HEI-OC1 cells. Kanamycin was applied at 100 *μ*M and furosemide at 10 *μ*M. Both concentrations were less than the concentrations that did not produce cytotoxicity in the CCK assay after 14 days (Figures 3(a) and 3(b)). Individual treatment with kanamycin or furosemide increased the response of Kir4.1. However, when the two drugs were administered together, a significant reduction in Kir4.1 expression was observed compared to the control (Figure 4(a)). NKCC1 showed a similar pattern to that of control in the presence of kanamycin, but expression was decreased in the presence of furosemide alone or the combination of kanamycin and furosemide (Figure 4(b)). Compared to control, the expression of KCNQ4 was decreased in the presence of kanamycin alone or in combination with furosemide. However, KCNQ4 expression was increased in the presence of furosemide alone compared to control (Figure 4(c)). Kanamycin blocks the KCNQ4 channel, and furosemide blocks the NKCC1 cotransporter. Thus, the results supported the anticipated result of a complementary role of K^+^ channels with ototoxic drugs. To confirm that Kir4.1 plays an important role in HEI-OC1 cells, Kir4.1 was completely inhibited by the transfection with 40 pmol siRNA (Figure 5(a)). Cell death did not occur in this concentration (Figure 5(c)). When treated with both 40 pmol of siRNA and ototoxic drug, Kir4.1 was completely inhibited (Figure 5(b)). In the absence of ototoxic drug, there was no effect on cell death, while cell death occurred in the presence of ototoxic drug (Figure 5(d)). The observations indicated the significant impact of the reduction of Kir4.1 on the survival of hair cells.

### 3.4. Changes of Kir4.1 in the Mouse Model of Ototoxicity

**Figure 6:**
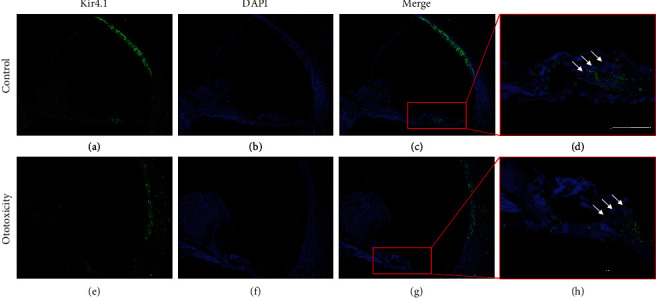
Changes in Kir4.1 expression in control and ototoxic mice. (a–d) Kir4.1 expression in control mice. (a) Expression of Kir4.1 in stria vascularis (StV) and outer hair cells (OHC) of control mice. (b) Pattern of staining with 4′,6-diamidino-2-phenylindole (DAPI). (c) Merged image. (d) High expression of Kir4.1 in OHC. (e–h) Kir4.1 expression in ototoxic mice. (e) Expression of Kir4.1 in StV and OHC of ototoxic mice. (f) Pattern of DAPI staining. (g) Merged image. (h) Organ of Corti displayed decreased intensity expression of Kir4.1 in OHC.

**Figure 7:**
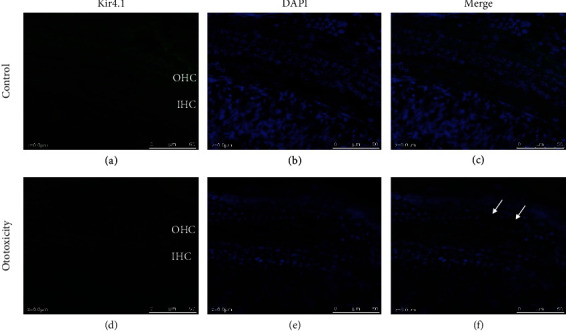
Changes in expression of Kir4.1 *ex vivo*. Kir4.1 expression in outer hair cells and inner hair cells (OHC) in cochlear explants of control and ototoxic mice. (a–c) Explant of the control mouse. (a) Kir4.1 is strongly expressed in OHC. (b) Upon DAPI staining, cell loss was observed. (d–f) Explant of ototoxic mouse. (d) The expression of Kir4.1 in OHC was decreased compared to the control. (e) Upon DAPI staining, cell loss was observed in OHC.

**Figure 8:**
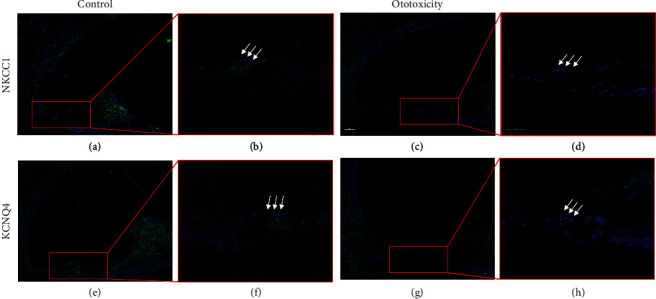
Changes of NKCC1 and KCNQ4 expression in control and ototoxic mice. The changes of NKCC1 and KCNQ4 expression in the outer hair cells (OHC), stria vascularis (StV), and spiral ganglion (SG) of control mice and ototoxic mice. (a–d) NKCC1 expression in control and ototoxic mice. (a) StV, OHC, and SG displayed strong expression of NKCC1. (b) Magnified image of the organ of Corti revealing the strong expression of NKCC1 in OHC. (c) NKCC1 expression of StV, OHC, and SG are decreased compared to control. (d) Magnified image of the organ of Corti revealing the significant decrease of NKCC1 in OHC. (e–h) KCNQ4 expression in an ototoxic mouse. (e) OHC and SG display strong expression of KCNQ4. (f) Magnified image of the organ of Corti revealing high-intensity fluorescence of KCNQ4 expressed in the cell body part of the OHC. (g) KCNQ4 expression in OHC and SG is decreased compared with control. (h) Magnified image of the organ of Corti revealing the significant decrease in fluorescence intensity of KCNQ4 in OHC.

Kir4.1 expression in the cochlea of the mouse ototoxic hearing loss model was confirmed upon treatment with the kanamycin-furosemide combination. Immunofluorescence assay results revealed the strong expression of Kir4.1 in the stria vascularis and outer hair cells in control mice. In contrast, mice with ototoxicity-related hearing loss displayed decreased expression of Kir4.1 in the stria vascularis as well as in outer hair cells (Figure 6).  Figure 7 shows the destruction of outer hair cells in ototoxic hearing loss model and decreased expression of Kir4.1. NKCC1 and KCNQ4 expressions were confirmed in the mouse model using the kanamycin-furosemide combination. In the immunofluorescence assay, NKCC1 and KCNQ4 were highly expressed in the stria vascularis and outer hair cells of the control mice, but the expressions in both locations were reduced in mice treated with the drug combination (Figure 8).

## 4. Discussion

**Figure 9:**
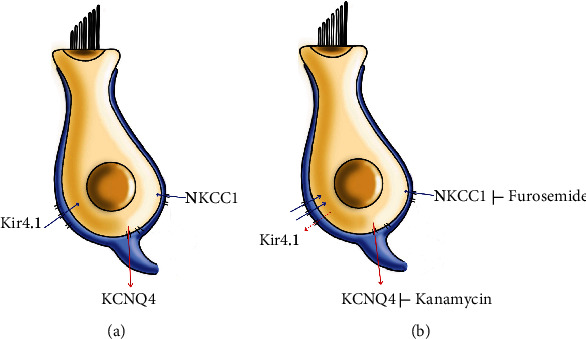
Correlation of Kir4.1, NKCC1, and KCNQ4 in HEI-OC1 cells. Correlation between the main potassium channels Kir4.1, NKCC1, and KCNQ4. When NKCC1 is blocked, the Kir4.1 channel has compensatory action and allows increased influx of potassium into cells. Kir4.1 is normally an inwardly rectifying K^+^ potassium channel. However, when the KCNQ4 channel is blocked, Kir4.1 channel allows efflux of K^+^ instead of through the KCNQ4 channel.

The presence of K^+^ in the endolymphatic circulation of the inner ear is important, since K^+^ is the major charge carrier for sensory transduction [26, 27]. The present data affirm the importance of the Kir4.1 in hair cells, one of the ion channels that is influential in maintaining the EP of the cochlea. Kir4.1 is also expressed in hair cells in addition to the stria vascularis in the inner ear. The changes in the correlation of Kir4.1 channels with the NKCC1 cotransporter and KCNQ4 channels during exposure to ototoxic drugs (kanamycin and/or furosemide) were demonstrated (Figure 9). Endolymphatic K^+^ flows into the sensory hair cells via the apical transduction channel and is released from the hair cells via basolateral K^+^ channels including KCNQ4 [11]. We previously described that a single application of kanamycin induced hearing loss in a mouse model incorporating furosemide [24]. The mechanism of ototoxicity was not definitely determined. Kanamycin inhibits the outward channel KCNQ4. When kanamycin was applied, Kir4.1 expression increased, which maintained the K^+^ concentration inside the cells. The Kir4.1 channel seems to have a role as an outward channel for the compensation of the K^+^ osmotic gradient. Although the Kir4.1 channel is an inwardly rectifying K^+^ channel, it can have an outward role in some cases [14, 28, 29]. Kanamycin treatment did not change the expression of NKCC1 from that of the untreated group. This is because the Kir4.1 channel has an outward role in place of KCNQ4, suggesting that the expression of the NKCC1 inward channel is similar to that of the untreated group. During treatment with furosemide, which blocks NKCC1, the expression of KCNQ4 increased. This is because K^+^ is released through the KCNQ4 channel, so the Kir4.1 channel only acts as an inward channel [29]. We suggest that the Kir4.1 channel has a key compensatory role in K^+^ circulation in hair cells when the NKCC1 cotransporter or KCNQ4 channel is injured.

Supportive cells are in fact glia cells. That is why Kir4.1 (the glia channel (Pooplalasundaram et al., 2000) [30]) is expressed in the glia-neuronal progenitors HEI-OC1. Such progenitors express the potassium inwardly rectifying channel Kir4.1 encoded by KCNJ10 gene which is the major channel that helps to keep healthy membrane potential in glial cells and supports not only K-buffering but also, most importantly, glutamate buffering by glia cells (Kucheryavykh et al., 2007) [31]. Glutamate buffering is extremely important to avoid the toxicity of glutamate excess.

The conditions for HEI-OC1 cell culture to assess cytotoxicity might be better at 33°C and 10% CO_2_ (the permissive condition for proliferation) than at 39°C and 5% CO_2_ (the nonpermissive condition for differentiation) [25]. In the nonpermissive condition, cells display similar characteristics of adult mouse hair cells in the inner ear. However, most researchers have selected the permissive condition because it permits an examination of whether cell death occurred due to spontaneous apoptosis or drug-related cytotoxicity. The present study developed the culture condition for the ototoxicity experiments during 5 days of growth at 33°C followed by 5 days at 39°C. The ion channels we analyzed in HEI-OC1 cells displayed different characteristics in the permissive and the nonpermissive conditions. In one study, Kir4.1 channels could induce cell maturation characterized by a shift of the cells from G2/M phase to the G0/G1 phase, which involved membrane hyperpolarization [32]. We presently utilized the nonpermissive condition for the experiments examining the ion channels of hair cells that were similar to adult differentiated hair cells. The K^+^ channels of the hair cells were typically more strongly expressed at 39°C than at 33°C. When cultured at 33°C for less than 5 days and then transferred to 39°C, Kir4.1 was not expressed constantly at 39°C. For this reason, we cultured the cells at 33°C for a certain period, shifted the cells to 39°C, and continued the cell culture.

The inward rectifier K^+^ channel family has more than 20 members. They comprise seven subtypes (designated Kir1.x–7.x) [33]. Kir4.1 in the cochlea is expressed only in the intermediate cells of the stria vascularis in the cochlear lateral wall [12, 34, 35]. Liu et al. [14] studied the Kir4.1 transcriptome, which reflects the genes that are being actively expressed in a cell, in the inner hair cells and outer hair cells of CBA/J mice. Kir4.1 had a relatively higher level of expression in both inner and outer hair cells. Jin et al. reported [36] that in guinea pig cochlea, KCNJ10 immunoreactivity was detected in strial intermediate cells, Deiters' cells, pillar cells in the organ of Corti, and spiral ganglion satellite cells. The observations are consistent with the view that although the role of Kir4.1 in the lateral wall of the cochlea is to facilitate the entry of K^+^ into the scala media, Kir4.1 of hair cells could have an important role in recycling K^+^ from the hair cells to the perilymphatic space [15]. To explore this possibility, we studied the presence of Kir4.1 in HEI-OC1 cells and found that the combination of kanamycin and furosemide directly affected ion channels in hair cells and caused ototoxicity. We screened ion channels that are commonly expressed in the kidney and inner ear. K^+^ channels were thought to be important in maintaining the endocochlear potential of the cochlea. Therefore, three K^+^ channels (Kir4.1, NKCC1, and KCNQ4) among the channels screened in HEI-OC1 cells were predicted to be mainly responsive to ototoxic drugs. We targeted these three channels. The ion and water transport functions in the inner ear help maintain the proper endolymph K^+^ concentration required for hair cell function. NKCC1 is also a K^+^ cotransporter protein that plays an important role in K^+^ recycling [11]. The survival of the outer hair cell critically depends on a specific K^+^ conductance mediated by KCNQ4 (Kv7.4) channels [37, 38]. The aminoglycoside antibiotics gentamicin and neomycin inhibit KCNQ4 channels in cochlear outer hair cells by depleting phosphatidylinositol [4, 5] bisphosphate [39]. Therefore, the Kir4.1, NKCC1, and KCNQ4 K^+^ channels were predicted to respond to ototoxic drugs. Hence, we targeted these three channels.

The single injection of the combination of kanamycin and furosemide is a novel technique for inducing ototoxicity in several models [24, 40, 41]. Abbas and Rivolta [41] used 400 to 500 mg/kg kanamycin, followed by an intraperitoneal injection of furosemide 100 mg/kg after 20 to 30 min in gerbils. Ju et al. [24] used various doses of kanamycin (420–600 mg/kg) with 130 mg/kg furosemide in C57BL/6 mice. Knowledge of the mechanism of ototoxicity induced by the combination of kanamycin and furosemide is important for an understanding of the maintenance of EP. However, the mechanism remains unclear. Presently, kanamycin (<1 mM) and furosemide (50 *μ*M) did not kill HEI-OC1 cells. The concentrations were determined based on experiments in mice. We think this was because of ion channel compensation. After treatment with siRNA to reduce KCNJ10 gene and, thus, disable the most important channel, cytotoxicity was confirmed by immediate response to the dose of ototoxic drugs. This suggests that the combined ototoxic drug directly affects hair cells and that Kir4.1 plays an important role in the compensation. When mice were treated with kanamycin and furosemide without any suppression of Kir4.1, hair cell death and hearing loss were observed. In vivo kanamycin and furosemide may also affect Kir4.1 in the stria vascularis, resulting in disruption of the balance of ion channels in the hair cells.

This study was limited as the use of HEI-OC1 cells does not reflect the complex structures of cochlea in animals. However, the use of HEI-OC1 cells did identify the importance of Kir4.1, suggesting a robust method for the ototoxicity of drugs. Kir4.1 was sufficiently expressed in the culture condition for ototoxicity experiments by a regimen involving cell growth for 5 days at 33°C followed by a shift to 39°C for 5 days. Kir4.1 in HEI-OC1 cells has a role in compensating for the balance of potassium transport with NKCC1 and KCNQ4. With the inhibition of Kir4.1, kanamycin 100 *μ*M and furosemide 10 *μ*M were lethal to cells. Although the expression of Kir4.1 was decreased mainly in the lateral wall of the cochlea in mice after injection of kanamycin and furosemide, Kir4.1 in outer hair cells was directly injured by the drugs. The decrease of Kir4.1 in hair cells rendered the cells vulnerable to death and hearing loss. Thus, if the decrease in Kir4.1 in hair cells can be prevented, it may be possible to prevent cytotoxicity caused by ototoxic drugs. This will require further study.

## Data Availability

All data generated or analyzed during this study are included in this published article.

## Abbreviations

HEI-OC1 cells: House ear institute-organ of Corti 1 cells
Kir4.1: Inwardly-rectifying potassium channel
RT-PCR: Reverse transcriptase PCR
qRT-PCR: Quantitative real-time PCR
Kan: Kanamycin
Furo: Furosemide
StV: Stria vascularis
OC: Organ of Corti
SG: Spiral ganglion
OHC: Outer hair cell
IHC: Inner hair cell
RM: Reissner membrane.

## Supplementary Materials

Supplementary Materials Supplementary Table 1: primer sequences.
