# Supplementary material for: Role of Kir4.1 Channels in Aminoglycoside-Induced Ototoxicity of Hair Cells
Source: Biomed Res Int. 2023 Dec 16;2023:4191999. doi: 10.1155/2023/4191999 (PMC10748730; doi:10.1155/2023/4191999)
Supplement: Supplementary Materials — Supplementary Table 1: primer sequences. [file 4191999.f1.pdf]

Supplementary Table 1. Primer Sequences

No.	Primer	Forward	Reverse	Length(bp)
1	Kir4.1	GGAGGAGATCCTCTGGGGTT	CCACTGGGAGATGCCACTTT	117
2	NKCC1	TAGGTCTCTCTGTCGTCGTA	AATAGTACGCTCCTCCTCCT	114
3	KCNQ4	CCCGGAAACCCTTCTGTGTC	AAAGATGAGCACCAGGAACC	245
4	Kcnmb1	CTGGGAGTGGCAATGGTAGTG	CCGAGTGTCTTCCGTGTGATAC	237
5	ClcnkA	GACCCTTCAGGCGCTGTTCGT	CGTAAACCGGGGTGAGATTTGTCC	330
6	ClcnkB	CTGGTGGGCGTTGTAAAAAGGAC	GGGAGGATTGGTCAGGGTTGAA	285
7	Scnn1A	GCACCCTTAATCCTTACAGATACACTG	CAAAAAGCGTCTGTTCCGTG	81
8	Scnn1B	CTCGGTGCTGTGCCTCATTG	GCCTCAGGGAGTCATAGTTGGG	278
9	Scnn1G	TGGTCCTCCTATCCTCGTTCTG	GTCACACCCATCAGGCAATAGC	344
10	Gja1	CGTTAAGGATCGCGTGAAG	GTTCTGCAAGCACCCTTTTT	508
11	Gjb6	CCCAAGTTAAGCACTTCAAGG	TTCAGCAATCGGCAATGTAT	611
12	Slc26A4	GACTGTAAAGACCCTCTTGATCTGA	GGAAGCAAGTCTACGCATGG	90
13	Atp2B1	CGGAAAATACAGGAGAGCTATGG	CTTTCCAAACACTGCTTCTCTTC	123
14	Atp6V1B1	AGGACAGTGTGCAGCGTCAAT	CCTGAACAATGGCCTTGGTC	151
15	Atp6V0A4	CAAATGTGAACAGCTTCCAGAGG	AGCATCCTTCTCAGGCACTTGG	131
16	β-actin	TTCTACAATGAGCTGCGTGTG	GGGGTGTTGAAGGTCTCAAA	122
